# Reply: Suspicions of possible vaccine harms must be scrutinised openly and independently to ensure confidence

**DOI:** 10.1038/s41541-020-0203-8

**Published:** 2020-07-06

**Authors:** Michael G. Head, Magdalen Wind-Mozley, Peter J. Flegg

**Affiliations:** 1 Faculty of Medicine, University of Southampton, Southampton, UK; 2 Independent Researcher, Newbury, UK; 3 Department of Medicine, Blackpool Teaching Hospitals NHS Foundation Trust, Blackpool, UK

We previously highlighted^1^ the complaint letter (dated 26 May 2016) addressed to the European Medicines Agency (EMA) by Jørgensen et al. and note the contents of their response^2^. We are grateful for their confirmation that they infer no causal link between HPV vaccine and neurological symptoms, such as complex regional pain syndrome (CRPS) and postural orthostatic tachycardia syndrome (POTS), as well as confirming it is their view that serious side effects from vaccines are rare. However, we disagree with some of their comments. We also note and wish to clarify that in their response, they refer to their own complaint which was then rejected by the ombudsman^3^. This complaint was published on 10th October 2016 and is not the letter to the EMA that we refer to in our article^1^.

Jørgensen et al. state in their correspondence to us that we claimed that the Danish Health and Medicines Authority report was based solely on case studies from Brinth et al.^2^. However, we did not state this^1^. They also dispute our opinion that the EMA review was wide-ranging. This review included coverage of clinical trial data, phase IV and pharmacovigilance studies and analysis of the case studies covering CRPS and POTS that the authors^2^ suggest are evidence of concern. It is still our opinion that the EMA’s review is wide-ranging, and that safety reviews in response to initial concerns are indeed acted upon by regulatory agencies exactly as the authors^2^ seem to request. Active ongoing pharmacovigilance is carried out by the EMA, and products are all subject to regular periodic safety assessment reports^4^. It is crucial that vaccine safety continues to be taken seriously by the regulatory agencies, but if the scientific community gives legitimacy to suspicions that are not warranted by the evidence, we do to the public and the medical profession a great disservice.

Jorgensen et al.’s^2^ submitted complaint to the European Ombudsman about the EMA review concerned issues such as transparency and openness, and its impartiality^5^. In October 2017, the Ombudsman concluded that the EMA’s conflict of interest policy was fully complied with, and that there was no evidence of maladministration on the part of the EMA during their review^3^.

Jorgensen et al.^2^ assert that allowing the pharmaceutical sector to carry out their own research to assess for harms is inappropriate. This overlooks the numerous public and charitably funded studies that have clarified the excellent safety profile of the vaccine—for example, several large studies have focused on the Denmark population^6–8^. As Jørgensen et al. describe^2^, HPV vaccine coverage in Denmark is now very low, with uptake of only around 15% in Danish girls born in 2004^9^. This shows the impact publicity surrounding very rare adverse events can have, and it is important now to rebuild trust in the HPV vaccine^9^. In 2017, the Danish Health Authority, the Danish Cancer Society and the Danish Medical Association launched the public campaign “Stop HPV, Stop Cervical Cancer,” in order to rebuild confidence in the HPV vaccine^10^.

Jorgensen, Jefferson and Gotzsche in 2018 wrote that a review highlighting the excellent safety profile of the HPV vaccine had ‘missed nearly half of the eligible trials’ and this showed ‘important evidence of bias’^11^. However, this claim is actually untrue^12^, and post-publication peer review shows that there are several further reported inaccuracies^13^. Jorgensen et al. say that we must not ‘jeopardise the trustworthiness of our profession’. We agree, and they should ensure that their comments in the peer-reviewed and lay literature are in future accurate and appropriate.
